# A Telerehabilitation Approach to Chronic Facial Paralysis in the COVID-19 Pandemic Scenario: What Role for Electromyography Assessment?

**DOI:** 10.3390/jpm12030497

**Published:** 2022-03-19

**Authors:** Alessandro de Sire, Nicola Marotta, Francesco Agostini, Vera Drago Ferrante, Andrea Demeco, Martina Ferrillo, Maria Teresa Inzitari, Raffaello Pellegrino, Ilaria Russo, Ozden Ozyemisci Taskiran, Andrea Bernetti, Antonio Ammendolia

**Affiliations:** 1 Department of Medical and Surgical Sciences, University of Catanzaro “Magna Graecia”, 88100 Catanzaro, Italy; alessandro.desire@unicz.it (A.d.S.); vera.dragoferrante@gmail.com (V.D.F.); inzitari@unicz.it (M.T.I.); ilaria.russo003@gmail.com (I.R.); ammendolia@unicz.it (A.A.); 2 Department of Anatomical and Histological Sciences, Legal Medicine and Orthopedics, Sapienza University, 00185 Rome, Italy; francescoagostini.ff@gmail.com (F.A.); andrea.bernetti@uniroma1.it (A.B.); 3 Sant’Anna Crotone Institute, 88900 Crotone, Italy; andreademeco@hotmail.it; 4 Department of Health Sciences, University of Catanzaro “Magna Graecia”, Viale Europa, 88100 Catanzaro, Italy; martinaferrillo@hotmail.it; 5 Antalgic Mini-Invasive and Rehab-Outpatients Unit, Department of Medicine and Science of Aging, University “G. d’Annunzio” of Chieti-Pescara, 66100 Chieti, Italy; raffaello.pellegrino@ucm.edu.mt; 6 Department of Physical Medicine and Rehabilitation, School of Medicine, Koç University, Istanbul 34010, Turkey; otaskiran@ku.edu.tr

**Keywords:** telerehabilitation, rehabilitation, telemedicine, digital health, remote medical diagnosis, digital transmission of medical care, precision medicine, shortwave diathermy, electromyography

## Abstract

There is a lack of data on patient and diagnostic factors for prognostication of complete recovery in patients with peripheral facial palsy. Thus, the aim of this study was to evaluate the role of a telerehabilitave enhancement through the description of a case report with the use of short-wave diathermy and neuromuscular electrical stimulation combined to facial proprioceptive neuromuscular facilitation (PNF) rehabilitation in unrecovered facial palsy, in a COVID-19 pandemic scenario describing a paradigmatic telerehabilitation report. A 43-year-old woman underwent a facial rehabilitation plan consisting of a synergistic treatment with facial PNF rehabilitation, short-wave diathermy, and neuromuscular electrical stimulation (12 sessions lasting 45 min, three sessions/week for 4 weeks). Concerning the surface electromyography evaluation of frontal and orbicularis oris muscles, the calculated ratio between amplitude of the palsy side and normal side showed an improvement in terms of movement symmetry. At the end of the outpatient treatment, a daily telerehabilitation protocol with video and teleconsultation was provided, showing a further improvement in the functioning of a woman suffering from unresolved facial paralysis. Therefore, an adequate telerehabilitation follow-up seems to play a fundamental role in the management of patients with facial palsy.

## 1. Introduction

Central facial palsy results from damage to the proximal nerve portion and epitomizes as a unilateral movement impairment, opposite to the side of the injury, with predominance in the lower face [1]. In contrast, peripheral facial palsy results from injuries to extratemporal elements of the nerve, for example, iatrogenic and oncological damage, inflammation such as herpes zoster (Ramsay Hunt syndrome), or an idiopathic condition, such as Bell’s palsy (BP) [2,3,4]. Its incidence ranges from 23 to 35 cases for 100,000, with a first peak at 30–50 years old and a second one in patients aged 60–70 years [5]. The etiology of facial palsy is still not adequately explained and could be due to several causes, including trauma, surgery, tumors, and viral infections (e.g., varicella-zoster, mononucleosis, herpes simplex virus) [6,7]. Facial palsy might be classified as acute, subacute (within 6 months of the onset), or chronic (>6 months), considering that the different evolution depends on the severity of the damage [8]. Among the clinical manifestations, patients with facial palsy might also have difficulty in closing the eye, resulting in dryness and abrasion of the horns, and oral incompetence caused by muscle weakness resulting in difficulty in eating and drinking [9]. Facial nerve palsy results in altered facial symmetry that might have a negative effect on health-related quality of life (HRQoL) by compromising communication and expression [10,11,12].

The diagnosis of facial palsy is commonly clinical, and there are several facial function classification systems proposed [13]. The severity of facial paralysis is commonly identified by the Sunnybrook facial grading system (SFGS) [14], evaluating symmetry at rest, symmetry during voluntary movements, and synkinesis. A variety of electrophysiological tests have been developed over the past years to measure the severity of nerve damage in patients with facial nerve palsy and temporomandibular disorders [15,16,17]. In this scenario, the best method for detecting degenerative damage to the facial nerve and also for detecting regeneration after nerve reconstruction is currently electromyography (EMG) of the facial muscles, derived with bipolar electrodes [18,19]. In this case, it is not the nerve itself that is examined but its target organ [20]. However, this method is not perfect either: nerve degeneration can only be detected 2 weeks after the onset of nerve damage, since Wallerian degeneration takes so long to reach the facial muscles [18]. Nevertheless, just with a surface EMG (sEMG), the cumulative action potential of voluntary activity is derived, which normally shows an interference pattern in all facial muscles, and any damage to the nerve results in a rarefication of the interference pattern [21].

To date, there is a wide variety of conservative therapeutical approaches, including antiviral drugs, corticosteroids, vitamin B12, acupuncture or dry needling, botulinum toxin, and rehabilitation [22,23,24]. Over the last century, several rehabilitative interventions have been described with inconsistent results in terms of improving function in facial palsy [25]. A Cochrane Systematic review [26] has recently shown that there is no high-quality evidence supporting any physical modality in patients with idiopathic facial paralysis. However, proprioceptive neuromuscular facilitation (PNF) combined with steroid treatment has been demonstrated to improve timing and recovery of injury, compared to medical therapy alone, in severe Bell’s palsy [2,27]. PNF is a rehabilitative technique of neuromuscular stimulation facilitation based on the activation or reactivation of proprioceptors using diagonal patterns; since most of the muscle fibers in the face run diagonally, it might be functional for the facial muscles [24,28,29,30]. In the last years, a high interest has been growing regarding the use of instrumental physical therapies, such as lower-level laser therapy and electrical stimulation, in improving facial muscle function in patients with facial palsy [30,31,32,33]. At the same time, short-wave diathermy (SWD) is considered as an innovative therapeutical option to reduce pain and improve HRQoL in musculoskeletal disorders [33]. Even if it is not recommended in the first phase of the acute viral inflammation, because it could worsen facial nerve edema and predispose to facial nerve degeneration [22], SWD determines pain relief and increases metabolic functions, preparing facial muscles for physical exercise [34].

Physical rehabilitation therapy is the measure most often prescribed by clinicians, but effective rehabilitation is often linked to the patient’s motivation and compliance with the rehabilitation process [35]. However, access to a specific facial rehabilitation protocol is also limited, so patients with facial palsy are advised to perform self-sufficient home exercise programs [36]. However, these physiotherapy exercises are often performed incorrectly, not as frequently as recommended, or stopped after some time [8].

Tan et al. [37] have recently compared video- to face-to-face assessment of the House–Brackmann grade, Sydney system score, and Sunnybrook system score, reporting similar reliability in all approaches. However, there was a poor reliability in the assessment of synkinesis, and 2D video lacked the anterior–posterior axis, which can potentially lead to biased evaluation of faces [38]. There are now commercially available 3D video cameras, which are important in judging faces in a three-dimensional fashion but remain high-cost and often inaccessible for a telerehabilitation context [38].

Like any technological approach, telerehabilitation has some advantages and disadvantages. In terms of benefits, home telerehabilitation systems are convenient if the intervention is used only to monitor or evaluate patients during facial complementary corrective therapy [39]. The possibility of staying in touch through telematic technologies allows patients with a serious facial deficit, such as severe cognitive deficits, to carry out physiotherapy at home without having to make demanding journeys. In terms of disadvantages, a problem could be the loss of human contact (face-to-face interaction) with the health worker. Furthermore, for each patient, system operators are required to optimize teletherapy according to the type of disease, and sometimes this is not possible due to high costs [40].

To date, in addition to clinical and imaging evaluation, instrumental functional evaluations are available through sEMG [19] but without evidence on the role of telemedicine.

Therefore, considering that digital patient technology would provide home therapy and a specialist facial recovery monitor, by this paradigmatic case report and literature review, we aimed at evaluating the role of a telerehabilitation approach in the treatment of a woman with chronic facial paralysis.

## 2. Case Presentation

A 43-year-old Caucasian woman presenting with chronic facial palsy was referred to a Physical Medicine and Outpatient Rehabilitation of a “Mater Domini” University Hospital of Catanzaro, Italy. In her medical history, she was referred to the emergency room on 31 August 2020 due to an asymmetry of the oral rhyme, with lower lip lowering on the right. She underwent a computed tomography (CT) scan of the head that was negative. She was diagnosed with a paralysis of the seventh cranial nerve of the right side by the physician that prescribed the following therapy: amoxicillin plus clavulanic acid (1 tablet for 3 times/day for 6 consecutive days), betamethasone (4 mg/day for 4 days), l-acetyl-carnitine (1000 mg 2 times/day for 6 days), and right eye patching.

In September 2020, the patient underwent a cranial magnetic resonance imaging (MRI) scan that showed a “tenuous impregnation in correspondence of the right internal auditory canal of the geniculate ganglion and intrapetrous portion of the seventh cranial nerve as from inflammation of the right facial nerve”. Then, in November 2020, the patient underwent another neurological examination that confirmed the diagnosis of right facial nerve palsy and suggested a physiatric evaluation.

However, considering the COVID-19 pandemic, the patient had some difficulties in referring to the hospital. Therefore, only in December 2021, she was admitted to our Physical Medicine and Outpatient Rehabilitation with a chronic right facial palsy. At the first clinical examination (T0, baseline), we observed a slight asymmetry of the buccal rhyme with a lowering of the lower lip on the right, especially exacerbated during specific movements: smiling, curling the lips, swelling the cheeks, flattening of the nasolabial fold on the right, closing the eyes, moving the lower eyelid, moving forehead muscles, and moving eyebrow.

Thus, after this clinical evaluation, we prescribed a specified facial rehabilitation plan consisting in a synergistic treatment with facial PNF rehabilitation, short-wave diathermy, and neuromuscular electrical stimulation, comprised of 12 sessions lasting 45 min, three sessions/week for a total of 4 weeks. Each session consisted of 30 min of facial PNF rehabilitation, aimed at facilitating the voluntary response of impaired facial muscles, manipulating the three main fulcra (superior, intermediate, and inferior) using both contralateral contractions and basic proprioceptive stimulation (stretching, maximal resistance, hand contact, and verbal input), 15 min of short-wave diathermy and low-frequency neuromuscular electrical stimulation performed through a bipolar hand-piece (Imperium 400, Brera Medical Technologies, Ogliastro Cilento, Salerno, Italy) [33] on the following muscles: corrugator, zygomaticus major, upper lip elevator, lower lip depressor, risorius, mirtiform, orbicularis oris, orbicularis oculi, chin, and frontal.

At the end of this intervention, the patient underwent another clinical evaluation (T1), and started a 1-month home-based rehabilitative protocol consisting of 15 min telemedicine sessions, two times/week. A physical therapist performed a video (see Supplementary Materials Video S1: The telerehabilitation approach to the patient with chronic facial paralysis) instructing the patient to perform the following specific home-based exercises: raising eyebrows, wrinkling the forehead, closing the eye, wrinkling nose, keeping a pen on the upper lip, chewing gum with left and right side of the mouth, keeping 20 mL of water in the left and right side of the mouth, making bubbles in a glass with a straw, puckering the lips, biting the lower lip, biting the upper lip, kissing paper, smiling with and without showing teeth, and moving the tongue to the right and left cheek. All the above-mentioned sessions were supervised by a physical therapist through videocalls. After this telerehabilitation program, we performed a follow-up clinical evaluation (T2).

**Figure 1:**
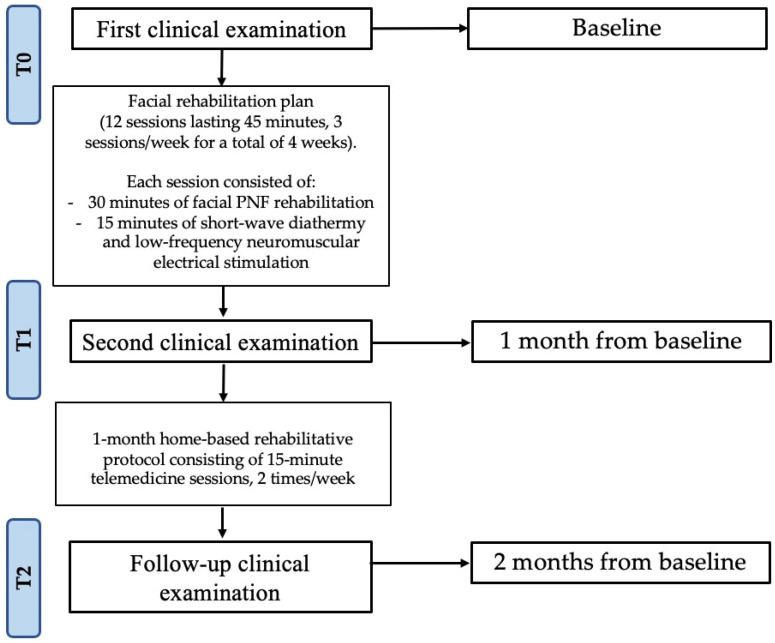
Study flow chart. Abbreviation = PNF, proprioceptive neuromuscular facilitation.

As the depicted in Figure 1, at all time-points (T0, baseline; T1, end of rehabilitative treatment; T2, end of telerehabilitation), the following clinical outcome measures were assessed: Sunnybrook facial grading system (SFGS), House–Brackmann (HB), facial nerve grading system, facial clinimetric evaluation (FaCE), synkinesis assessment questionnaire (SAQ), visual analog scale (VAS), Beck depression inventory (BDI), hospital anxiety and depression scale (HADS), symptom checklist-90 (SCL-90), EuroQol-5 Dimension 3 Level scale (EQ-5D-3L index), and EuroQol visual analog scale (EQVAS) [13,41].

The sEMG was performed with a wireless EMG device (FREEEMG 1000, BTS Bioengineering, Milan, Italy) using bipolar surface electrodes (diameter, 0.8 cm; interelectrode distance, 2 cm; pre-gelled disposable, surface Ag/AgCl Ambu^®^ Neuroline 720 electrodes (Ambu^®^, Neuroline, Ballerup, Denmark) [42,43]. Before performing the sEMG, the surface of the skin was shaved, gently abraded, and cleaned with alcohol to reduce the impedance of the skin at all time-points (T0, T1, and T2). The electrodes were placed according to SENIAM guidelines [44] on the frontalis muscle and orbicularis oris of both sides. The sEMG was performed during three maximum bilateral facial contractions, obtained through simple and standardized verbal commands: “raise your eyebrows” for wrinkles on the forehead, “close your eyes”, “smile”, “show your teeth”, “purse your lips” [19].

At the baseline (T0), the patient showed a severe facial palsy (SFGS = 25), with a moderate dysfunction (HB scale = Grade 3 and facial nerve grading system 2.0 = Grade III) and a moderate impairment and disability associated with facial dysfunction (FaCE = 59). At the end of the rehabilitative treatment (T1), the patient improved in all of the outcome measures (Table 1).

The SFGS score improved from 25 to 51; the facial nerve grading system from Grade III to Grade II; the HB scale improved from moderate to mild dysfunction (grade 3 vs. grade 2); the FaCE score improved from 59 to 67. The patient showed a reduction of HADS anxiety and depression (from 6 to 3 and from 5 to 2, respectively) and an improvement of HRQoL (EQ-5D-3L: 0.688 to 1; EQVAS: 70% to 90%).

**Figure 2:**
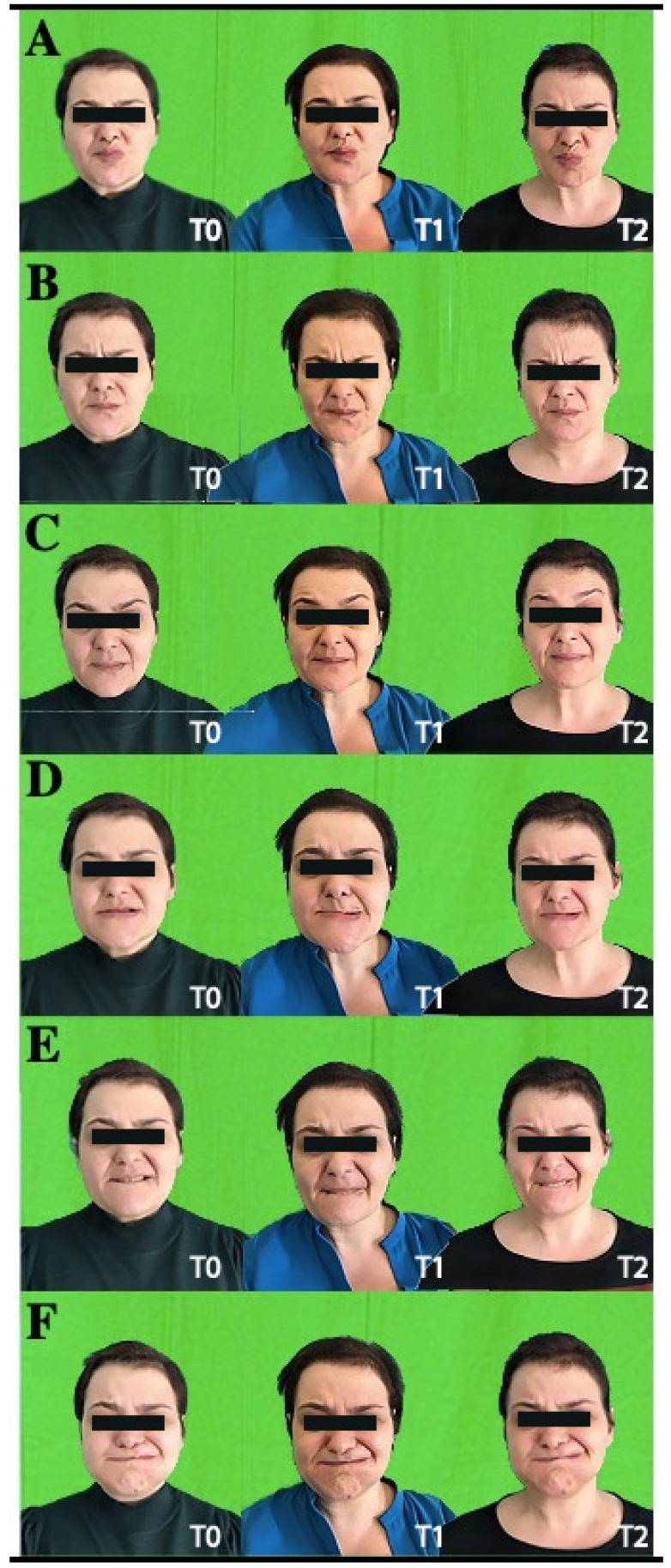
Movements executed by the patient during the examination to evaluate facial palsy at the different time-points from the left to the right: T0 (baseline), T1 (after 5 weeks), and T2 (at the end of treatment). (**A**): kiss with closed lips; (**B**): wrinkle the forehead; (**C**): elevate eyebrows; (**D**): bite the upper lips; (**E**): bite the lower lips; (**F**): puff up the cheeks.

After the telerehabilitation program (T2), the patient maintained a mild facial palsy; the SFGS improved from 51 to 73, and SAQ improved from 44.4% to 33.3%; HADS anxiety improved from 3 to 2; depression scores decreased from 2 to 1, and EQVAS improved from 90% to 100% (see Figure 2 for further details).

**Figure 3:**
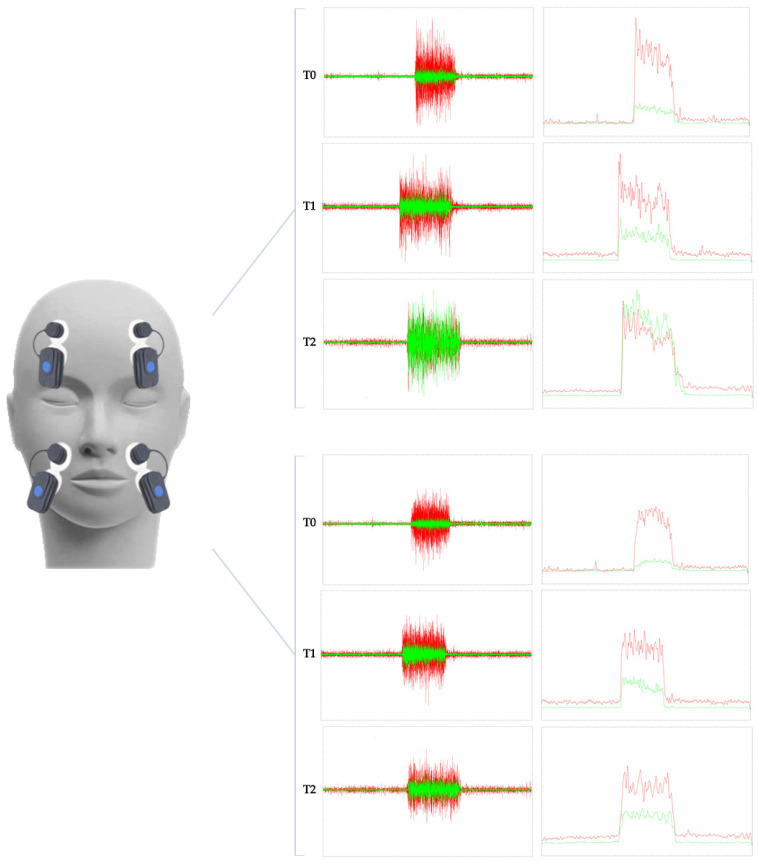
The graph shows the sEMG signal of the frontal muscle in the upper portion of the figure and the orbicularis oris in the lower portion of the figure. EMG representations are outlined in green for the side of the palsy and red for the side of the face not affected by the paralysis. At each timepoint of the evaluation of the case report, the raw data are represented on the left, while on the right the envelope of the sEMG signal is filtered and rectified to evaluate the ratio between the two mean amplitudes. So, for both muscles at T0, there is an obvious difference between paralysis and the normal side, while, at T1 and T2, the difference between the left and right-side decreases.

Concerning the sEMG evaluation of facial muscle activity, the ratio calculated on the signal envelopes [45,46] between palsy side and normal side, showed an improvement from T0 to T1 of the frontal muscle (T0 = 0.26 versus T1 = 0.45) and orbicularis oris muscle (T0 = 0.19 versus T1 = 0.31). Moreover, muscle activity of the upper face assessed by sEMG showed noteworthy differences at 1 month after the telerehabilitation approach (frontal muscle (T1 = 0.45 versus T1 = 0.91) and orbicularis oris muscle (T0 = 0.31 versus T1 = 0.47) (Figure 3)).

## 3. Discussion

The aim of this study was to evaluate the role of telerehabilitation as a valid, reproducible, and continuative facial rehabilitation program, taking into account the recent scientific literature and describing a paradigmatic case of a woman with chronic facial palsy having undergone combined short-wave diathermy and PNF technique followed by remote rehabilitative protocol. At T0, there was a consistent electromyographic difference between palsy side and normal side, despite the pharmacological therapy prescribed from the symptoms’ onset. As a starting point, we utilized sEMG to objectively evaluate the degree of muscle excursion and symmetry of voluntary movement and monitor the results of the rehabilitation protocol proposed [19].

In line with the study of Pavese et al. [47], there is often a relevant delay between symptom onset and rehabilitative program that could affect the outcomes of the intervention. Indeed, it is recommended to start an early rehabilitation program with a special focus on education of the patient, soft tissue mobilization to improve facial muscle stiffness and edema, functional retraining including neuromuscular reeducation, and synkinesis management [33]. When this does not happen and palsy overcomes the acute phase, one possible strategy is represented by short-wave diathermy and neuromuscular electrical stimulation, which has shown interesting results in enhancing muscle strength, improving muscle atrophy, and reducing spasticity in chronic facial palsy [29]. The synergic intervention described by our group in a previous study [33] demonstrated a significant improvement in symmetry of voluntary movement in unrecovered Bell’s palsy, with a mean improvement in the SFGS from 30.3 ± 7.8 to 47.2 ± 10.90. On the other hand, we registered a greater improvement in the functional scales administered (SFGS from 25 to 73) that could be explained by the combination of physical therapy and PNF. According with Monini et al. [2], patients receiving PNF based on proprioceptive neuromuscular facilitation showed a faster and higher improvement after treatment compared with patients treated only with medical therapy. PNF consists in the facilitation of the voluntary response of an impaired muscle applying a resistance.

The second phase of the rehabilitation program continued in a telerehabilitation setting, which is considered a treatment and evaluation modality that represents an effective extension to conventional rehabilitation, especially during the COVID-19 pandemic [40,48]. The patient instructed by the physical therapist through Skype videocall has maintained the results obtained at T1, showing a further improvement in some of the scores shown in Table 1. Telerehabilitation is a hospitalization avoidant approach, focusing on home-based rehabilitation deployment and integrating the treatment in daily life activities with minimal resources and little direct supervision, that could be a valid therapeutic choice in facial palsy, especially for those patients who are unable to attend the hospital setting regularly [39]. Finally, facial palsy, independently from the etiology, is associated with anxiety and depression, especially in women with a worsening of quality of life [48]; thus, psychosocial consequences of a facial palsy should always be considered in terms of HRQoL.

In general, access to a specialized therapist is limited, and, unfortunately, the effectiveness of any facial rehabilitation is commonly related to the intensity and frequency of each session [3]. Therefore, facial therapy for patients with facial palsy is usually associated with home training [49]. However, the patient’s compliance with this home training has not been sufficiently deepened so far, but today the barriers to this approach are often insurmountable: adaptation of exercises to daily life, use of a mirror, and lack of regular feedback from a therapist [50]. The discussion of such forms of training, including telerehabilitation, is acquiring a whole new meaning due to the COVID-19 pandemic [40]. As in the present study, patients with more severe impairment of facial expression and psychosocial impairment rated significantly higher acceptance with these innovative systems [35].

Furthermore, the need for telerehabilitative adaptations to exercises can challenge patients’ motivations, but, for the physiotherapist on the other hand, he can provide immediate feedback to the therapeutic plan in teleconsultation, as, among other things, it is an opportunity to see the patient immersed in his own [51] domestic context, making it possible to analyze emotional and environmental factors that can mitigate barriers and identify facilitating components [52].

sEMG is the best evaluation tool to characterize a whole facial muscle and to evaluate inter-muscular coordination. More in detail, the sEMG is non-invasive and painless, and the number of probes used, and, therefore, the number of facial muscles analyzed simultaneously, is unlimited. Moreover, sEMG allows a detailed analysis of the coordinated muscle activation needed for specific tasks and emotional expressions [21].

In this context, a simple instrumental diagnosis might help the clinician in the management of these patients. By decoupling the kinematic investigation and relying on just four surface EMG probes, the findings of this case report might provide us a simple but crucial tool for monitoring these patients, even in a telehealth scenario [19].

Since facial palsy is managed and treated only in highly specialized medical facilities, several patients have access problems, mainly due to distance, time constraints, mobility, and frequency of visits [53]. Their physician could envision a telemedicine setting so that patients might receive the same standard of care from home, minimizing the number of face-to-face assessments [54]. Moreover, these problems, in combination with the current COVID-19 crisis, result in a growing demand for electronic/mobile health applications and, with rapidly increasing communication technology, make it more possible than ever [55]. For this reason, the use of even a video (Supplementary Materials Video S1) would provide an accessible and simple protocol to these patients, in order to provide therapeutic continuity in the pandemic context, but also to all those people with facial palsy who do not have the possibility to access the hospital environment regularly.

Given the results of our study’s approach to telemedicine, health practitioners should support and encourage the development of telemedicine, including with policies and protocols, facilitating its accessibility to healthcare professionals [54]. With the COVID-19 pandemic, telemedicine could become an integral part of medical education, not underestimating developments in the protection of privacy, but above all by developing more accessible strategies for health professionals in order to increase the care of these patients with chronic disorders [55,56].

To the best of our knowledge, there is a lack of evidence on the combined effect of physical therapy, PNF, and telerehabilitation in facial nerve palsy that could represent a milestone on the treatment of unrecovered facial palsy.

## 4. Conclusions

Taken together, an objective evaluation based on sEMG alone could lead to an easy diagnostic strategy, guiding the physician on appropriate treatment. Moreover, a combined and continuative rehabilitative treatment including SWD, neuromuscular electrical stimulation, PNF, and telerehabilitation improved facial muscle functionality, psychological, and social disability in a woman affected by facial palsy.

## Figures and Tables

**Table 1:** Clinical outcome measures.

Clinical Outcome Measures	T0	T1	T2
Sunnybrook Facial Grading System	25	51	73
HB Scale	Grade 3: Moderate dysfunction	Grade 2: Mild dysfunction	Grade 2: Mild dysfunction
Facial Nerve Grading System 2.0	Grade III: Moderate dysfunction	Grade II: Mild dysfunction	Grade II: Mild dysfunction
FaCE	59	67	69
SAQ Worksheet	55.6%	44.4%	33.3%
VAS	1	0	0
BDI	4	1	1
HADS			
*HADS-A*	6	3	2
*HADS-D*	5	2	1
SCL-90			
*Somatization*	5	7	0
*Obsessive-Compulsive*	1	1	0
*Interpersonal Sensitivity*	1	0	0
*Depression*	3	1	0
*Anxiety*	4	1	0
*Hostility*	1	0	0
*Phobic anxiety*	0	0	0
*Paranoid ideation*	0	0	0
*Psychoticism*	0	0	0
*Sleep disorders*	4	6	2
EQ-5D-3L	0.689	1.000	1.000
EQVAS	70%	90%	100%

Abbreviations: HB = House–Brackmann; FaCE = facial clinimetric evaluation; SAQ = synkinesis assessment questionnaire worksheet; VAS = visual analog scale; BDI = Beck depression inventory; HADS = hospital anxiety and depression scale; SCL-90 = symptom checklist-90; EQ-5D-3L = EuroQol-5 Dimension 3 Level scale; EQVAS = EuroQol visual analog scale.

## Data Availability

Dataset is available on request.

## Supplementary Materials

The following supporting information can be downloaded at: https://www.mdpi.com/article/10.3390/jpm12030497/s1, Video S1: The telerehabilitation approach to the patient with chronic facial paralysis.
